# Supplementary material for: COZOID: contact zone identifier for visual analysis of protein-protein interactions
Source: BMC Bioinformatics. 2018 Apr 6;19:125. doi: 10.1186/s12859-018-2113-6 (PMC5889581; doi:10.1186/s12859-018-2113-6)
Supplement: Supplementary file 2 — Software build. Executable binary file of the software tool. (ZIP 118,784 kb) [file 12859_2018_2113_MOESM2_ESM.zip › cozoid/platform/docs/testng-6.5.1-javadoc.zip!/org/testng/IInstanceInfo.html]

IInstanceInfo (TestNG)


---


|  |  |  |
| --- | --- | --- |
| **Overview** \| **Package** \| **Class** \| **Tree** \| **Deprecated** \| **Index** \| **Help** | |  |
| **PREV CLASS**   **NEXT CLASS** | **FRAMES**    **NO FRAMES**     **All Classes** |  |
| SUMMARY: NESTED \| FIELD \| CONSTR \| METHOD | DETAIL: FIELD \| CONSTR \| METHOD |  |


---


## org.testng Interface IInstanceInfo

---

``` public interface IInstanceInfo ```

This class defines a pair of instance/class. A method with @Factory
can return an array of these objects instead of Object[] so that
instances can be dynamic proxies or mock objects and still provide
enough information to TestNG to figure out what classes the
annotations should be looked up in.

**Author:**
:   Cedric Beust

---

| **Method Summary** | |
| --- | --- |
| `java.lang.Object` | `getInstance()` |
| `java.lang.Class` | `getInstanceClass()` |

**Method Detail**

### getInstance

```
java.lang.Object getInstance()
```

:   **Returns:**: The instance on which the tests will be invoked.

---


### getInstanceClass

```
java.lang.Class getInstanceClass()
```

:   **Returns:**: The class on which the TestNG annotations should be looked for.


---


|  |  |  |
| --- | --- | --- |
| **Overview** \| **Package** \| **Class** \| **Tree** \| **Deprecated** \| **Index** \| **Help** | |  |
| **PREV CLASS**   **NEXT CLASS** | **FRAMES**    **NO FRAMES**     **All Classes** |  |
| SUMMARY: NESTED \| FIELD \| CONSTR \| METHOD | DETAIL: FIELD \| CONSTR \| METHOD |  |


---
